# Commentary: Effectiveness of telemedicine interventions on blood pressure control and self-management efficacy in hypertensive patients: a systematic review and meta-analysis

**DOI:** 10.3389/fpubh.2026.1788360

**Published:** 2026-04-01

**Authors:** Xueyan Jiang

**Affiliations:** Community Health Service Center of Xiangfu Street, Gongshu District (Gongshu District Hospital of Integrated Traditional Chinese and Western Medicine), Hangzhou, China

**Keywords:** health behavior, hypertension, medication adherence, meta-analysis, telemedicine

## 1 Introduction

The systematic review and meta-analysis by Yang et al. (1) provides a timely and valuable synthesis of evidence on the role of telemedicine in managing hypertension. The study's key strength lies in transcending the narrow focus on physiological endpoints by incorporating diverse outcome indicators, including behavioral and psychological constructs such as hypertension knowledge, self-efficacy, and self-management capacity, thereby offering a more comprehensive picture of telemedicine's holistic effects. Furthermore, the research demonstrates methodological rigor through PROSPERO pre-registration, adherence to PRISMA guidelines and Cochrane Handbook standards, and subgroup analyses exploring heterogeneity across different populations and intervention characteristics, providing important evidence based guidance for future research and practice. While the findings support the potential of telemedicine, a critical examination reveals several methodological and interpretive limitations that warrant discussion to strengthen the evidence base and guide future research and implementation.

## 2 Unexplored intervention heterogeneity threatens practical interpretation

A core challenge in synthesizing telemedicine research lies in the profound heterogeneity of interventions, which range from passive text messages to integrated platforms combining monitoring, automated feedback, and human coaching. While the authors employed subgroup analyses based on broad modality categories, this approach may not capture the critical “active ingredients” responsible for clinical effects. These components may refer either to specific functions of telemedicine, such as real-time monitoring and asynchronous communication, or to the integrated practices of hypertension management, including lifestyle guidance and medication management strategies. While the study notes that “various core components may be effective,” it fails to clarify whether the technological delivery mode or the management content plays the dominant role. As a result, the pooled estimates average across different components, obscuring the key drivers of success. Notably, the analysis found no significant between-group differences across intervention types (education, monitoring with feedback, comprehensive support). This suggests that the key to effective intervention may lie not in the technological format itself, but in its ability to successfully implement proven core practices in hypertension management. Future meta-analyses in this field would benefit from employing component network meta-analysis or detailed meta-regression to disentangle the effects of individual intervention characteristics, such as feedback personalization level or the presence of clinical oversight (2).

## 3 Clinical significance requires context beyond average reductions

The reported mean systolic blood pressure reduction of 4.62 mmHg is appropriately contextualized with population-level risk estimates. However, the clinical meaning of this reduction can vary significantly across individuals. A critical gap is the lack of analysis based on baseline risk stratification. For a patient with stage 3 hypertension, this reduction, while beneficial, may be insufficient, whereas for a patient with high-normal blood pressure, it might be highly meaningful. Furthermore, the review does not report the proportion of patients achieving guideline-recommended blood pressure targets, which is a more direct measure of success in clinical practice. Highlighting these distinctions would provide a more nuanced understanding of for whom telemedicine is most clinically impactful (3).

## 4 Overlooking the digital divide risks exacerbating health inequities

The finding of greater efficacy among younger patients and in certain regions inadvertently highlights a major ethical and practical challenge: the digital divide. Although the review noted regional differences in effectiveness, it inadequately addressed how socioeconomic status, digital literacy, age-related technological barriers, or cultural acceptability influence engagement and outcomes. More critically, this divide exists not only at the individual level but also profoundly at the health system level. In resource-constrained settings, lack of essential infrastructure (stable connectivity, remote monitoring devices), policy support, reimbursement mechanisms, and capacity to integrate telemedicine into existing workflows may constitute macro-level barriers to implementation. By systematically analyzing neither individual-level attrition rates nor health system-level adoption obstacles, the review missed a crucial opportunity to discuss how multidimensional equity-oriented design could narrow rather than inadvertently widen health disparities (4).

## 5 Insufficient integration of theory limits mechanistic understanding

Although the review mentions behavioral theories like social cognitive theory, it does not leverage the included studies to investigate how telemedicine works. Were interventions based on explicit theoretical frameworks? Which behavioral change techniques were most commonly associated with success? A more mechanistic analysis, involving the coding of intervention components against established taxonomies and linking them to outcomes, could move the field from asking “does it work?” to “how does it work best?” This theory-informed approach is crucial for designing more effective and efficient interventions rather than merely scaling complex, multi-component bundles (5).

## 6 Discussion

The meta-analysis by Yang and colleagues robustly confirms the potential of telemedicine in improving hypertension management. Its focus on behavioral outcomes and exploration of heterogeneity provides important references for future research. However, to translate this potential into equitable and effective real-world practice, the field must mature beyond demonstrating average efficacy. The limitations discussed point to necessary evolutions in research practice. Future studies should prioritize standardized reporting of intervention components to allow for finer-grained synthesis. They must embed equity-focused analyses, tracking recruitment, engagement, and outcomes across diverse demographic subgroups. Finally, grounding intervention design and evaluation in behavioral science theory will be key to optimizing their impact and understanding their mechanisms. Addressing these challenges will ensure that the promise of telemedicine is realized broadly and effectively, for all patients.
